# Diagnostic pitfall: primary myoepithelial carcinoma of the lacrimal gland, case report and literature review

**DOI:** 10.1186/s12907-018-0073-4

**Published:** 2018-08-02

**Authors:** Youssef Mahdi, Mohamed Amine Azami, Rajae Daoudi, Nadia Cherradi

**Affiliations:** 1 Department of Pathology, Specialties Hospital, Ibn Sina University Hospital, Rabat, Morocco; 2 Faculty of Medicine and Pharmacy, Mohammed V University of Rabat, Villa 670, Sala Al Jadida, Salé, Morocco; 3 Department of Ophtalmology, Specialties Hospital, Ibn Sina University Hospital, Rabat, Morocco

**Keywords:** Myoepithelial carcinoma, Lacrimal gland, Immunohistochemistry

## Abstract

**Background:**

In lacrimal gland, lymphomas and inflammatory lesions predominate. Primary epithelial tumours represent less than 30% of lacrimal gland lesions. Myoepithelial carcinoma of lacrimal gland is rare. To the best of our knowledge, only nine cases have been reported in the literature. This lesion presents diagnostic difficulties: non-specific clinical and radiological findings and histological polymorphism. This is well illustrated by the diagnostic pathology errors described in the literature.

We report a new case of lacrimal myoepithelial carcinoma with a review of others published cases to try to assess clinico-pathological features and outcome whenever possible of this rare tumour.

**Case presentation:**

An 80-year-old Arabian female presented with a 2-month history of swelling over the right eyebrow, pain, proptosis of the right eye and diplopia. Computed tomography demonstrated an ill-defined, homogeneous, contrast-enhancing mass attached to the medial rectus. A biopsy was performed. Microscopic examination showed malignant spindle cells tumour, most consistently to sarcoma or sarcomatoid carcinoma. Immunohistochemical study was not possible because neoplastic material has been exhausted. Subsequently, total exenteration of the right orbit was performed. Immunohistochemical study revealed diffuse positive staining for pancytokeratin AE1/AE3, epithelial membrane antigen (EMA) and smooth muscle actin (SMA) and focal positivity for S100 protein. The lesion was immunonegative for desmin, h-cladesmon, CD34, Melan-A and HMB-45. The tumour was extending to the surgical margins. The patient was lost to follow-up until she developed local tumour progression 3 months after removal. The patient was again lost to follow-up and therefore did not receive any other treatment in our hospital.

**Conclusion:**

We present this rare tumour with an unusual location. The use of a complete immunohistochemical panel with epithelial and myoepithelial markers positivity helped us for classification of this poorly differentiated tumour.

## Background

Lacrimal gland lesions represent 9% of all space-occupying orbital lesions [1]. Lymphomas and inflammatory lesions predominate [2, 3]. Primary epithelial tumours represent less than 30% of lacrimal gland lesions [2, 4]. Pleomorphic adenoma is the most frequent, accounting for approximately 50% of epithelial tumours [2, 4–6]. Adenoid cystic carcinoma is the most frequent malignant tumour [4–6].

Myoepithelial carcinoma of lacrimal gland is rare. To the best of our knowledge, only nine cases have been reported in the literature [6–14]. This lesion presents diagnostic difficulties. The clinical and radiological findings are not specific. The tumour is characterized by histological polymorphism. This is well illustrated by the diagnostic pathology errors described in the literature.

We report a new case of lacrimal myoepithelial carcinoma with a review of others published cases to try to assess clinico-pathological features and outcome whenever possible of this rare tumour.

## Case presentation

**Fig. 1:**
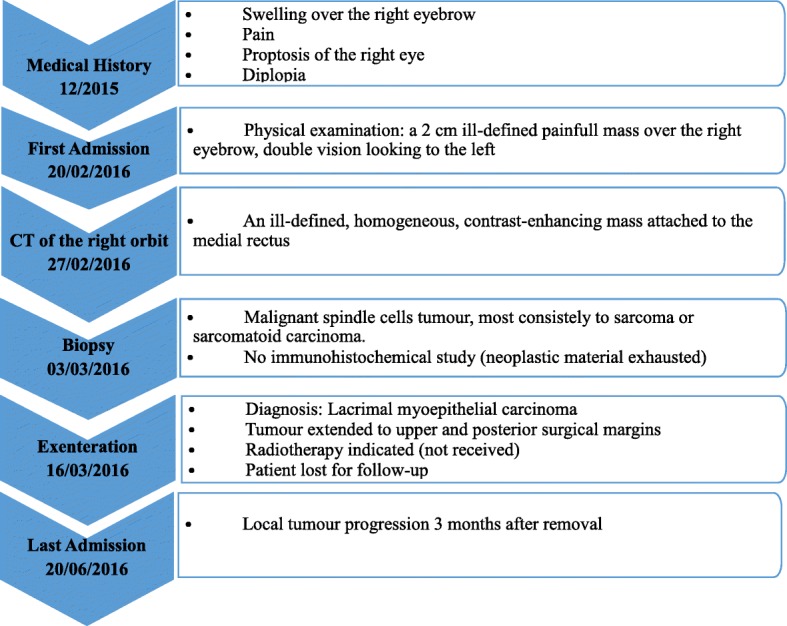
Case report timeline

**Fig. 2:**
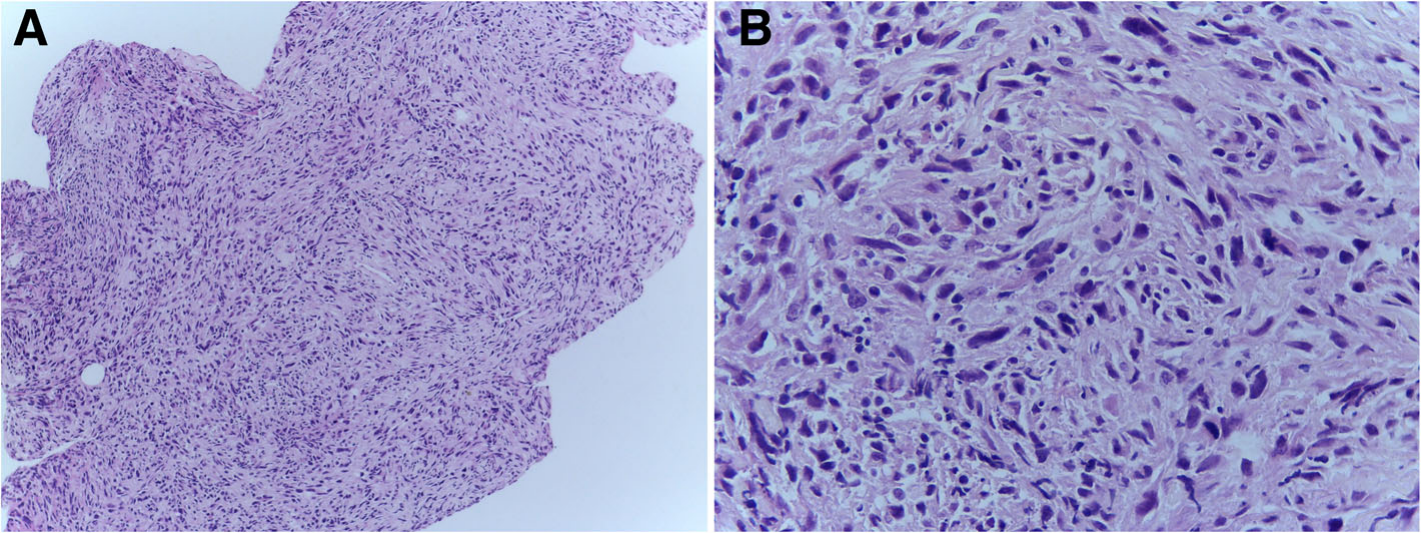
Histological aspect of the lesion in biopsy: **a** The tumour is composed of interlacing bundles of spindle cells (H&E × 100). **b** The neoplastic cells show anisokaryosis and hyperchromatic nuclei (H&E × 400)

**Fig. 3:**
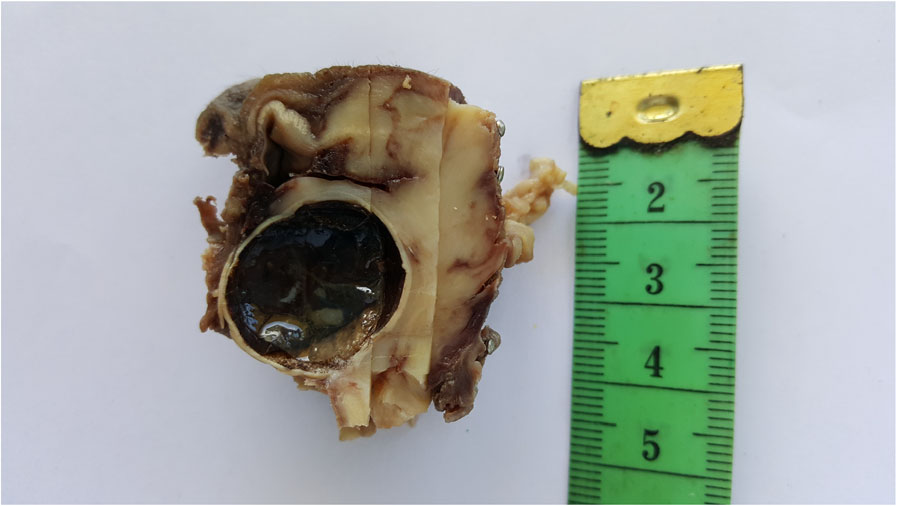
Gross features of the exenterated orbital contents: Cut surface shows ill-defined, whitish and firm tumour. It is attached to the sclera without infiltration into eyeball. It infiltrates the upper eyelid. The tumour is extending to the surgical margins

**Fig. 4:**
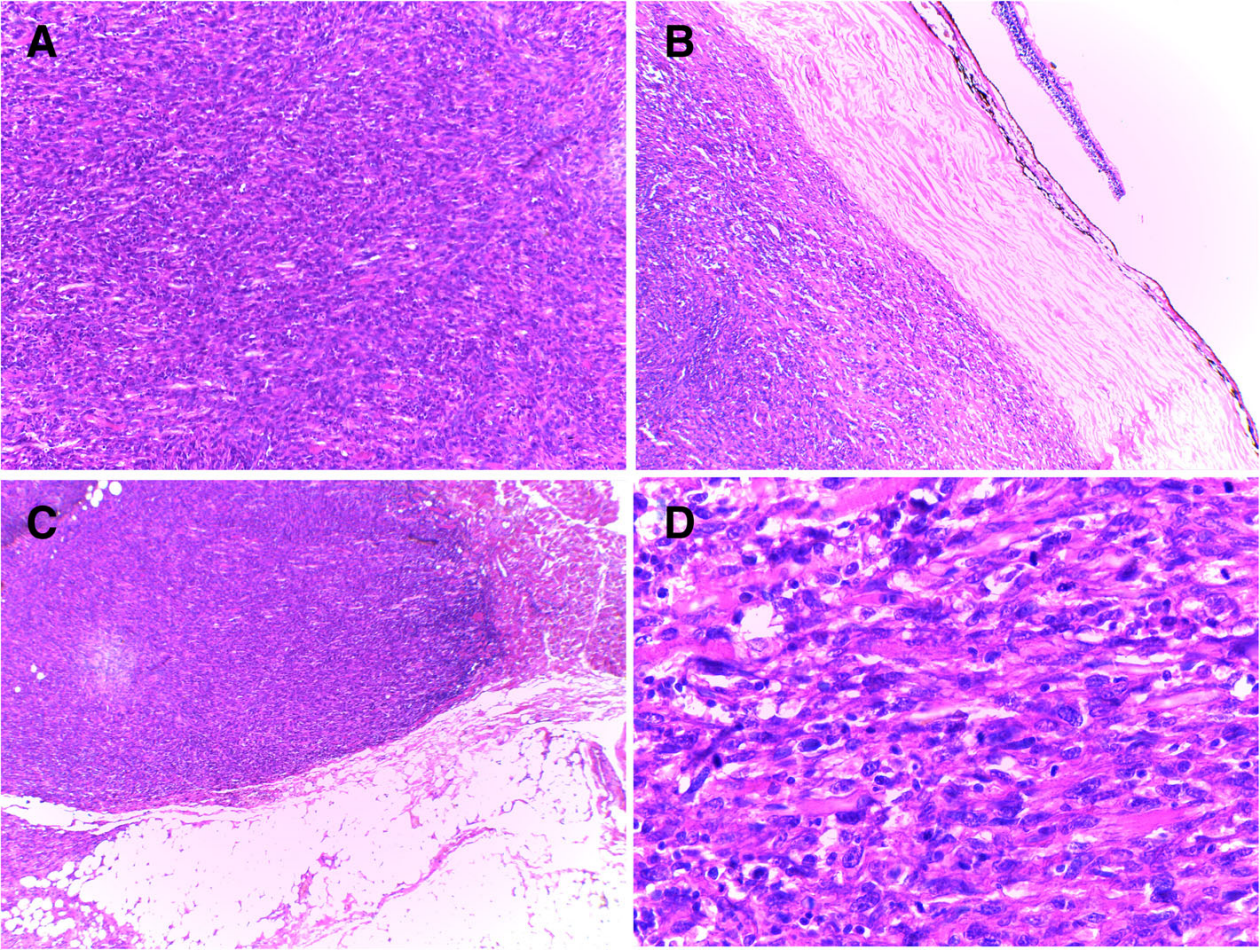
Histological aspect of the tumour after orbital exenteration: **a** Neoplastic spindle cells are arranged in disorganized fascicules (H&E × 100). **b** The tumour is attached to the sclera without infiltration (H&E × 40). **c** Infiltration into adipose tissue and striated muscle is observed (H&E × 40). **d** High-power view shows the obvious cytologic atypia and several mitoses of the neoplastic cells (H&E × 400)

**Fig. 5:**
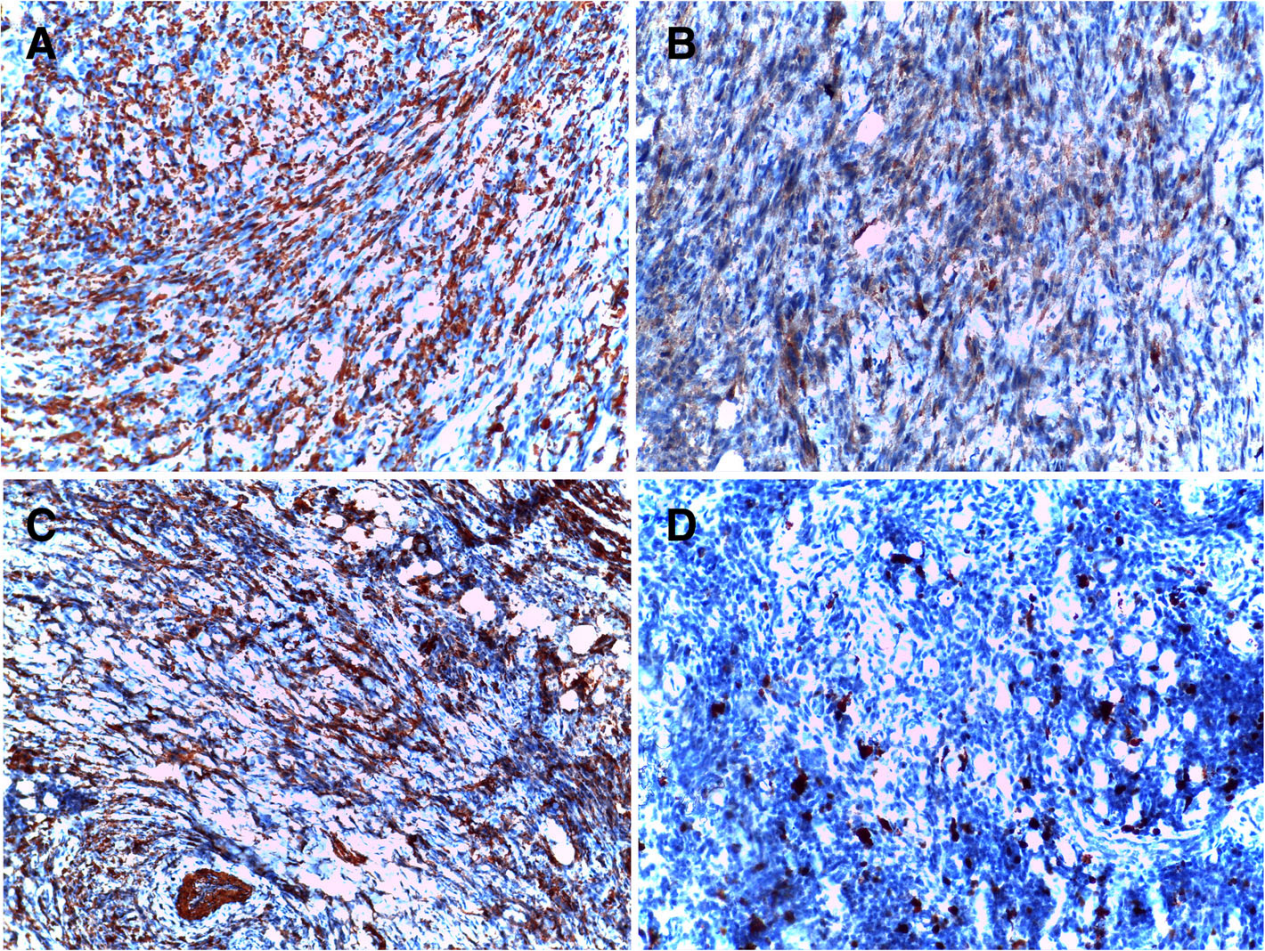
Immunohistochemical profile of the tumour after orbital exenteration: The tumor cells express pancytokeratin AE1/AE3 **a**, EMA **b**, SMA (**c**) and express focally S100 protein (**d**)

**Fig. 6:**
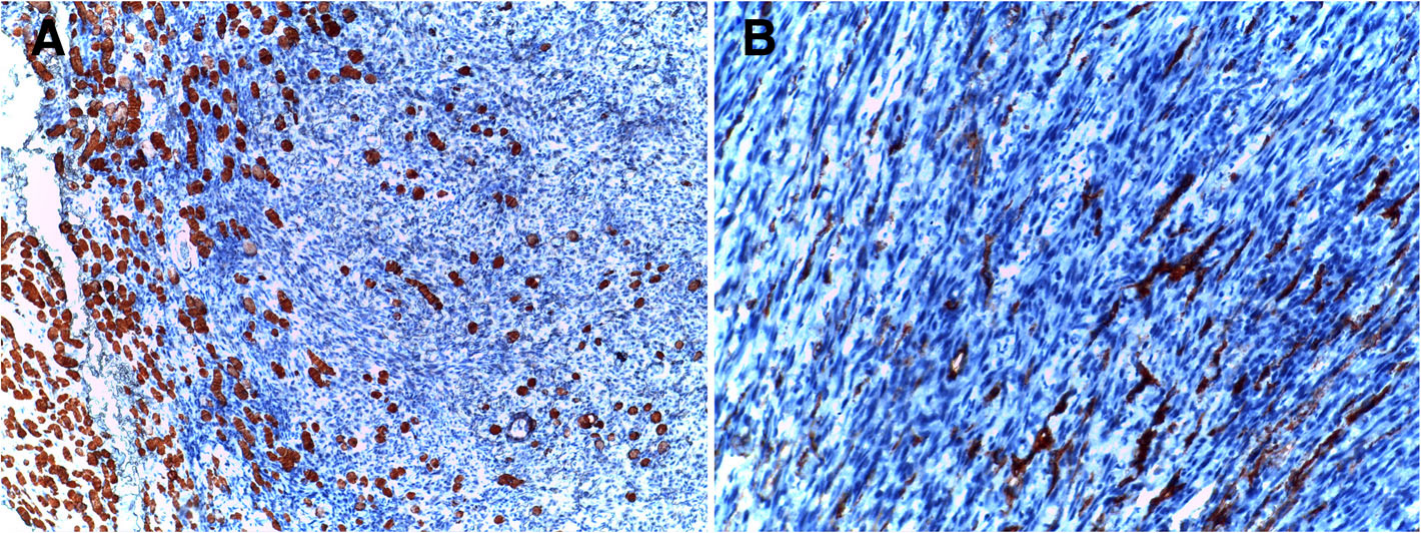
Immunohistochemical profile of the tumour after orbital exenteration: The tumor cells are negative for desmin (**a**) (infiltrated skeletal muscle fibers are staining with desmin) and CD34 (**b**) (vessel endothelial cells are staining with CD34)

An 80-year-old Arabian female presented to our hospital with a 2–month history of swelling over the right eyebrow, pain, proptosis of the right eye and diplopia (Fig. 1). Physical examination revealed a 2 cm ill-defined painful mass over the right eyebrow. The patient complains of double vision looking to the left. Computed tomography (CT) of the right orbit demonstrated an ill-defined, homogeneous, contrast-enhancing mass attached to the medial rectus. As a space-occupying orbital lesion, a lymphoma or a sarcoma was suspected. As a result, a biopsy was performed. On microscopic examination, the tumour was composed of interlacing bundles of spindle cells with anisokaryosis and hyperchromatic nuclei (Fig. 2). Some mitotic figures were observed. Immunohistochemical study was not possible because neoplastic material has been exhausted. The conclusion was malignant spindle cells tumour, most consistently to sarcoma or sarcomatoid carcinoma. No lymph node or distant metastases were found. Subsequently, total exenteration of the right orbit was performed under general anaesthesia. Dilute adrenaline was injected to lessen bleeding generally abundant in this type of excision. Periosteum was incised right around the orbital rim and separated from the bone passing back towards the orbital apex. The eyeball, eyelids, appendages of the eye and periosteum were removed. Surgical specimen was addressed for pathological examination. At gross examination, the tumour appeared ill-defined, whitish and firm. It measured 4/2.5/1.5 cm. It was attached to the sclera without infiltration into eyeball. It infiltrated the upper eyelid (Fig. 3). Microscopic examination revealed spindle cells forming disorganized fascicles. They have an irregular nucleus with vesicular chromatin and an eosinophilic cytoplasm. The mitotic index was 18 per 10 high-power fields. Adipose tissue and striated muscle infiltration was observed (Fig. 4). Immunohistochemical panel used for initial work up of this high grade spindle cell neoplasm was desmin, smooth muscle actin (SMA), pancytokeratin AE1/AE3, epithelial membrane antigen (EMA), S100 protein and CD34. This panel served to rule out first leiomyosarcoma, rhabdomyosarcoma, sarcomatoid carcinoma and spindle cell melanoma. It revealed diffuse positive staining for pancytokeratin AE1/AE3, EMA and SMA and focal positivity for S100 protein (Fig. 5). The lesion was immunonegative for desmin and CD34 (Fig. 6). As pancytokeratin AE1/AE3, EMA, SMA and S100 protein staining was positive, we completed by second panel. It showed h-cladesmon, Melan-A and HMB-45 negative staining. These pathological and immunohistochemical findings suggested the diagnoses of myoepithelioma, epithelial myoepithelial carcinoma and myoepithelial carcinoma. Myoepithelioma was excluded because the tumour borders were infiltrative. Epithelial myoepithelial carcinoma is by definition composed of a biphasic arrangement of inner luminal ductal cells and outer myoepitheliaI cells. However, the tumour did not show bi-layered duct-like structures. The most appropriate diagnosis was myoepithelial carcinoma. The tumour was extending to upper and posterior surgical margins. Radiotherapy was then indicated. The patient was lost to follow-up until she developed local tumour progression 3 months after removal. The patient was again lost to follow-up and therefore did not receive any other treatment in our hospital.

## Discussion

In our case there was no obvious attachment to lacrimal tissue. But, according to Shields JA et al. [15, 16], the lacrimal gland is the only structure where epithelial cells are encountered in the orbit. Therefore, this primary myoepithelial carcinoma was considered to have its origin in the lacrimal gland.

The clinical and radiological findings of lacrimal myoepithelial carcinomas were described with details in only six cases in the literature [8–10, 12–14] (Table 1). These features were not specific. The lesion size varied from 1.6 to 4 cm.

**Table 1:** Clinical and radiological findings in cases of lacrimal myoepithelial carcinomas described in the literature

Cases	Age-sex	Clinical presentation	Radiologic findings
Weis et al. [6]	Not reported	Not reported	Not reported
Herrera et al. [7]	Not reported	Not reported	Not reported
Iida et al. [8]	77 year-old, man	Proptosis	Not reported
Wiwatwongwana et al. [9]	84 year-old, man	Proptosis, Severe decreasing of vision Ocular pain Eyeball displacement	CT: a 3.2/2.6/2.2 cm well-circumscribed, calcified lacrimal gland mass extending to the apex, displacing the globe with irregularity in the adjacent bony orbital wall
Argyris et al. [10]	39 year-old, woman	Proptosis	CT and MRI: a 3/2.2/2 cm extraconal mass effacing the lacrimal grand and displacing the left lateral rectus, optic nerve and globe
Von Holstein et al. [11]	Not reported	Not reported	Not reported
Moret et al. [12]	88 year-old, man	Proptosis Decreasing of vision Lateral rectus muscle paralysis	MRI: a 3.5/2.5/1.7 cm intra- and extra-conal mass, extending to the lacrimal gland and the lateral rectus muscle
Rabade et al. [13]	27-year-old, man	Proptosis Decreasing of vision Swelling over the eyebrow	MRI: a well-defined, lobulated, contrast-enhancing mass in the superolateral compartment of the orbit with erosion of the lateral wall and roof and extending into the right frontal region
Larbcharoensub et al. [14]	68-year-old, woman	Proptosis Mass in the superior temporal part of the orbit Visual acuity of no light perception	MRI: a 3.8/3.7/3.3 cm well-defined, lobulated, vivid inhomogeneous enhancing isosignal T1W/slightly hypersignal T2W mass. It located at retrobulbar portion involving extraconal-conal-intraconal spaces of the orbit and invading of the lateral bony wall
Case presented (Mahdi et al.)	80 year-old, woman	Proptosis Pain Swelling over the eyebrow Diplopia	CT: an ill-defined, homogeneous, contrast-enhancing mass attached to the medial rectus

*CT* Computed tomography, *MRI* magnetic resonance imaging

The tumour occurred de novo [6, 8, 12] or on a pre-existing pleomorphic adenoma or adenoid cystic carcinoma [9, 10, 13, 14]. De novo lacrimal myoepithelial carcinoma would have a poorer prognosis when compared to lacrimal myoepithelial carcinoma arising from pleomorphic adenoma or adenoid cystic carcinoma.

Diagnoses discussed before pathological examination were not specified neoplasms, pseudo-tumours, hemangioma, lymphoma, sarcoma or metastatic process.

For the histological classification of epithelial lacrimal gland tumours, the World Health Organization (WHO) classification of salivary gland tumours is currently applied [17]. Indeed, the lacrimal and salivary glands present histological and pathological similarities. In addition, the WHO classification of these rare lacrimal gland tumours has not changed since 1980 [18].

At the salivary glands, WHO defines myoepithelial carcinoma as a malignancy entirely composed of neoplastic myoepithelial cells with an infiltrative growth [19]. The neoplastic cells can take on various morphological appearances: spindle, epithelioid, plasmacytoid or clear. In our case, the tumour was very cellular composed of spindle-shaped cells and resembled sarcoma. The tumour cells are most commonly arranged in solid, trabecular or reticular patterns [19]. Central necrosis and pseudocyst formation may occur [19]. Infiltrative and destructive growth is the major histological feature associated with malignant behavior [19].

Immunohistochemistry is required for the diagnosis. It demonstrates the myoepithelial nature of neoplastic cells. The diagnosis requires reactivity for cytokeratin and at least one of myoepithelial markers: SMA, glial fibrillary acidic protein (GFAP), calponin, S-100 protein, p63 and CK 5/6 [6–10, 12–14, 19]. No genetics studies were performed in the reported cases.

Due to histological polymorphism and rarity of the lesion in the lacrimal gland, myoepithelial carcinoma poses significant challenges in differential diagnosis. This is well illustrated by the diagnostic errors described in the literature (Table 2).

**Table 2:** Diagnostic pathology errors in cases of lacrimal myoepithelial carcinomas described in the literature

Cases	Diagnostic pathology errors
Weis et al.	Adenoid cystic carcinoma
Argyris et al.	Carcinoma ex-pleomorphic adenoma
Moret et al.	Biopsy: Leiomyosarcoma
	Exenteration: Epithelial-myoepithelial carcinoma
Case presented (Mahdi et al.)	Sarcoma/Sarcomatoid carcinoma

Weiss et al., in a retrospective multicenter cohort study of 118 cases of epithelial neoplasia found one case of myoepithelial carcinoma [6]. It was initially diagnosed as malignant epithelial neoplasm, most consistent with adenoid cystic carcinoma. It was composed of nests of epithelioid and clear cells with nuclear atypia. The immunohistochemical study showed positive staining for keratin, S-100 protein and actin.

In the case reported by Argyris et al. [10], no immunohistochemical stains were initially performed and the tumour was diagnosed as carcinoma ex-pleomorphic adenoma. After tumour recurrence, review of the histologic sections was done. The neoplasm was composed of an admixture of adenoid cystic carcinoma and poorly differentiated component expressing pancytokeratin AE1-AE3, CK 5/6, EMA, p16, SMA, S-100 protein, calponin and p63.

In another case, the tumour was composed exclusively of spindle-shaped cells [12]. It infiltrated the right lateral rectus and lacrimal gland. It had SMA positive staining at immunohistochemistry. It was diagnosed as leiomyosarcoma and surgical excision was indicated. The lesion showed spindle cells proliferation with a positive immunostaining for SMA and P63 and bi-layered duct-like structures. The diagnosis was epithelial-myoepithelial carcinoma, which is a low-grade tumor. Surveillance was recommended. Locoregional recurrence was observed 3 months after removal. The tumour was reclassified as myoepithelial carcinoma after histological review. The bilayered structures were entrapped non-neoplastic acinar structures.

In addition, Rabade et al. reported the case of tumour composed exclusively of clear cells [13]. It infiltrated surrounding bone trabeculae and striated muscle. These findings could make the diagnosis of clear cell carcinoma-not otherwise specified (NOS). Positive immunostaining with calponin identified the myoepithelial differentiation.

The sarcoma-like feature in the orbit can also evoke sarcomatoid carcinoma. It is positive for pancytokeratin AE1/AE3 and P63 but lakes S100 protein expression.

Surgical resection was the principal treatment in the reported cases. It consisted to wide local excision or exenteration of the orbit. No distant metastasis were found. In three cases, local or locoregional recurrences were diagnosed between 3 and 24 months [10, 12, 13]. Tumour cells were found in the resection margins [10, 12]. One of these three patients received palliative radiotherapy and died 8 months after diagnosis [12]. Another patient died within months of diagnosis without definitive treatment [6]. These two patients died from de novo myoepithelial carcinoma [6, 12]. One patient with myoepithelial carcinoma arising in pleomorphic adenoma presented three uneventful years without any adjuvant therapy [14]. Additional cases are needed to better characterize primary lacrimal myoepithelial carcinoma.

## Conclusions

We present this rare tumour with an unusual location. Given recurrence and death reported in patients with lacrimal myoepithelial carcinoma, correct diagnosis is imperative. The use of a complete immunohistochemical panel with epithelial and myoepithelial markers positivity helped us for classification of this poorly differentiated tumour.

## Abbreviations

CT: Computed tomography
EMA: Epithelial membrane antigen
GFAP: Glial fibrillary acidic protein
MRI: Magnetic resonance imaging
NOS: Not otherwise specified
SMA: Smooth muscle actin
WHO: World Health Organization
